# The Nice Nursing Home

**Published:** 1888-03-24

**Authors:** 


### The Nursing Mirror.

Communications for this column should be addressed The Mirror, care of the Editor, 38, Old Jewry, London, E.C.

### THE NICE NURSING HOME.

Having been a subscriber to your interesting journal since its publication, and appreciating in common with many others the honourable and liberal principles upon which it is conducted, I was surprised to find that you had published in this week's issue of The Hospital a letter which, in my opinion, is of too personal a character—evidently the outcome of petty spite—for insertion in a journal such as yours. I refer to your correspondent's letter re the "Nice Nursing Institution." Fortunately I am able to write disinterestedly, as I have never met either the present superintendent or her predecessor. This letter is calculated to be prejudicial to the reputation of the lady—whose system of management or personal character it is intended to condemn—as well as the Institution itself. Superintendents are too much at the mercy of unscrupulous persons, whom they may have had occasion to correct—and, however reluctantly, to make an example of perhaps—as it is; but The Hospital will indeed be opened with trepidation and not with pleasure if, through its agency, such personal "stabs" may be inflicted, and nurses warned, as it were, not to enter any particular institution where there has been "a change of superintendents," whose "ways" have not met with the approval of one, or it may be several, prejudiced or insubordinate persons. Of course anyone with common sense, if contemplating making an application to the "Nice Home," will not hesitate about doing so, in spite of this sweeping condemnation, until they have found out for themselves whether there is any truth in it or not. F. M.

Birmingham.

[*⁎* We publish this letter with pleasure, and also the one following it, which, as will be seen, is from the Lady Directress at Nice. We believe the best way to make unfounded charges fail is to let the actual facts be fully stated.—Ed. *T. H.*]

